# The Nanodiffraction beamline ID01/ESRF: a microscope for imaging strain and structure

**DOI:** 10.1107/S160057751900078X

**Published:** 2019-02-22

**Authors:** Steven J. Leake, Gilbert A. Chahine, Hamid Djazouli, Tao Zhou, Carsten Richter, Jan Hilhorst, Lucien Petit, Marie-Ingrid Richard, Christian Morawe, Raymond Barrett, Lin Zhang, Roberto A. Homs-Regojo, Vincent Favre-Nicolin, Peter Boesecke, Tobias U. Schülli

**Affiliations:** a ESRF – The European Synchrotron, 71 Avenue des Martyrs, 38000 Grenoble, France; b Aix Marseille Université, CNRS, Université de Toulon, IM2NP UMR 7334, 13397 Marseille, France

**Keywords:** X-ray, full-field microscopy, scanning microscopy, Bragg diffraction, coherent diffraction imaging

## Abstract

The ID01 beamline has been built to combine Bragg diffraction with imaging techniques to produce a strain and mosaicity microscope for materials in their native or *operando* state. A scanning probe with nano-focused beams, objective-lens-based full-field microscopy and coherent diffraction imaging provide a suite of tools which deliver micrometre to few nanometre spatial resolution combined with 10^−5^ strain and 10^−3^ tilt sensitivity. A detailed description of the beamline from source to sample is provided and serves as a reference for the user community. The anticipated impact of the impending upgrade to the ESRF – Extremely Brilliant Source is also discussed.

## Abstract 2

The ID01 beamline has been built to combine Bragg diffraction with imaging techniques to produce a strain and mosaicity microscope for materials in their native or *operando* state. A detailed description of the beamline from source to sample is provided and serves as a reference for the user community.

## 1. Introduction   

Since the discovery of their electromagnetic wave nature, the potential of the small wavelength of X-rays for microscopy has intrigued scientists. The high frequencies of X-rays being far above most resonant energies of bound electrons in matter makes refraction a very weak phenomenon. This imposes a severe absorption limit to the physically possible aperture of refractive lenses for X-rays. Even though today refractive, reflective and diffractive optics are steadily being developed, the situation cannot be compared with visible-light optics; the wavelength as a resolution limit, due to large-aperture optics with almost 100% transparency, is far from being reached. Although being less suited for serving microscopy, X-rays revealed the atomic structure of matter more than 100 years ago, marked by the Nobel Prize for Physics of 1914 awarded for the discovery of the diffraction of X-rays by crystals to Max von Laue. X-rays have since proved an unrivalled tool for the interrogation of crystal structures ranging from metals to molecules and the structure of life. The high sensitivity to the structure of matter is reflected by the absolute lattice parameter resolution obtained by X-ray diffraction. Traditionally X-ray diffraction was considered to have relatively poor spatial resolution yielding only spatial averages as useful results. The beamline ID01 at ESRF – The European Synchrotron has been conceived in order to exploit and combine the lattice parameter resolution supplied by X-ray diffraction with the resolution available in direct space by current imaging techniques using the most advanced X-ray optics technology. ID01 represents a microscope capable of imaging strain and structure often without sample preparation. The penetration power of X-rays makes this imaging tool compatible with sample environments for *in situ* studies and *operando* experiments on functional devices. The diffraction imaging techniques developed at this instrument can be divided into scanning techniques, full-field techniques and coherent reconstruction techniques. To supply a competitive tool for all these techniques the design of the beamline was optimized for beam stability on the one side and flexibility of the energy range and focusing mode on the other side. This requires different operation modes that begin with a minimum of optical elements and allow for a set of optional beam-tuning devices that cover a wide range of available X-ray energies, beam sizes and fluxes. In the following section the source and primary optics setup is presented followed by a detailed description of the endstation together with some benchmark values and examples.

## 2. Beamline overview   

**Figure 1:**
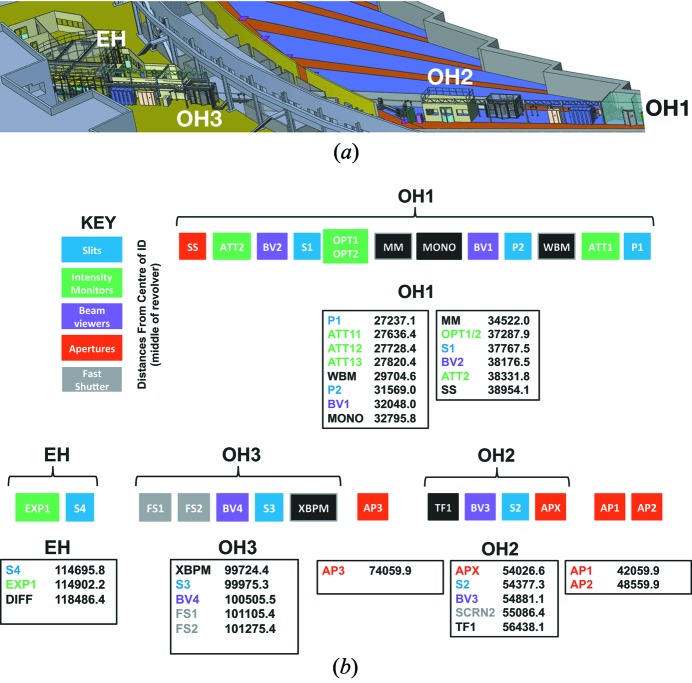
(*a*) Sketch of the three optics hutches and the experimental hutch of ID01. OH1 contains the principle beam-conditioning components such as monochromators and mirrors, OH2 contains the transfocator, OH3 contains the secondary source and EH contains the focusing optics. (*b*) Positions of all vital components which touch the X-ray beam, in millimetres from the source.

With a total length (source–detector) of 125 m ID01 represents one of the five longest beamlines of the ESRF, optimized for producing highly focused X-ray beams, see Fig. 1(*a*) ▸. It consists of three optics hutches, a primary hutch (OH1) ranging from 26 m to 40 m, a secondary hutch (OH2) for lenses for beam tuning at 55 m and a tertiary optics hutch (OH3) hosts a secondary source at 100 m. The experimental hutch with its two endstations extends from 116 to 125 m. The secondary source and the endstation are located on a highly stable concrete slab, specially designed and realized for the ESRF upgrade. Fig. 1(*b*) ▸ depicts all beamline components and their location relative to the X-ray source (defined as the middle of the storage ring straight section of ID01).

The principal elements of the beamline are described by following the optical path from source to endstation.

### 2.1. The X-ray source   

**Figure 2:**
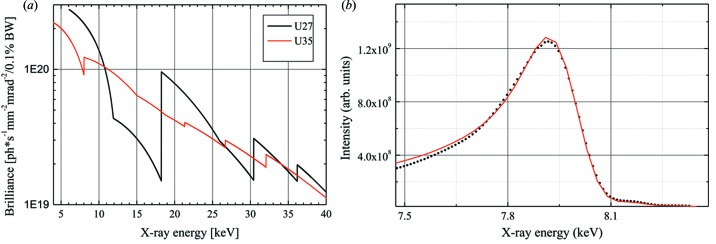
(*a*) Source brilliance at ID01 as delivered from the U27 and the U35 undulators. (*b*) Theoretical undulator lineshape for the U27 device at 8 keV (full line), compared with measurement (black dots). The measurement was performed in the monochromatic beam using a polyimide scattering foil and a photodiode.

The X-ray source of ID01 originates from up to three undulators, a single 27 mm period (U27), a single 35 mm period (U35) and a revolver which offers either of the aforementioned periods. Each can be operated at minimum gaps of 11 mm, limited by the vacuum chamber. The resulting brilliance optimum from the fundamental line of the U27 lies in the energy regime from 6.5 to 11 keV. This can be considered as the core energy regime for coherent diffraction techniques and also to some extent for scanning probe techniques. The third harmonic of the U27 yields a second brilliance maximum in the 19.5–22 keV regime. The U35 serves to assure the availability of the complete energy range and, in particular, to fill the gap between the first and third harmonic of the U27. At 8 keV, the source size amounts to 12 µm (V) × 120 µm (H) (FWHM) at a beam divergence of 20 µrad (V) × 170 µrad (H) (FWHM). The brilliance available from this configuration is presented in Fig. 2(*a*) ▸. For a typical X-ray energy, the line shape of the fundamental emission from a 1.6 m-long U27 device has been characterized and is presented in Fig. 2(*b*) ▸ (full dots) compared with the theoretical line shape (full line) and shows the undulator quality.

### 2.2. Primary optics and monochromators   

The first element in the optics hutch is a pair of high-power slits to shape the white beam as a function of required illumination of the optics. The illumination parameters can require a minimum slit size in order to clean the beam and limit the heat load on all optical elements to minimize thermal deformation when only a small fraction of the beam is needed, such as for coherent diffraction. A minimum beam size is also necessary to fully illuminate the first optical elements to ensure a homogeneous illumination and hence minimize thermal gradients on the optics. These primary slits are at a position of 27 m from the source, closest to the concrete shielding of the storage ring. Downstream a set of high-power diamond absorbers are installed. Such devices are typically used to reduce risk during alignment or to reduce heat load in general by suppression of the fundamental line when working on the third harmonic. In order to preserve the high brilliance in the vertical plane and ensure stability, the reflective optics (mirrors and monochromators) were chosen to reflect in the horizontal plane, a choice other nanoprobes have made as well (Paterson *et al.*, 2011 ▸). This is of particular interest for mirrors that work at grazing angles of total external reflection. Slope and figure errors present in such mirrors can impact the beam quality (wavefront) and lead to a loss of brilliance. In order to preserve the brilliance and avoid blurring the source size, the effect of these errors on the beam propagation direction should be lower than the angular source size. The inherent asymmetry of the source size (12 µm in the vertical and 120 µm in the horizontal) at the ESRF strongly favours the horizontal deflection geometry in the case of a mirror. The grazing angle geometry leads to geometric corrections that make such an optic significantly more tolerant to mirror slope errors in the sagittal than in the meridional direction. A simple geometric consideration leads to a correction factor of the impact of any mirror deviation of 1/sinθ for the meridional direction and of sinθ for the sagittal direction (Susini, 1995 ▸), θ being the angle of incidence. With a much higher tolerance of the horizontal source size, it is thus wise to use the more critical meridional mirror plane to coincide with the horizontal plane of the laboratory frame.

The main drawback of this geometry is an increase in the required length of the mirrors. The considerable horizontal divergence leads to beam sizes at the mirror position of 7 mm. The ID01 mirrors were chosen to be 900 mm long, collecting at an angle of incidence of 3–5 mrad over a wide energy range, 30–60% of the full beam intensity. The four available stripes, Si, Rh, Pt and the 6.3 nm periodic W/B_4_C multilayer, cover the energy range from 4 to 45 keV, see Table 1 ▸.

Beyond mirror slope- and figure errors, thermal effects caused by the white beam may effect the ideal mirror shape. The first mirror must tolerate the full power of the white-beam deformation due to the heat load, which has to be considered. To minimize these deformations the mechanical design of the mirrors was optimized with finite-element modelling, which confirmed that a complete illumination of the mirror is important in order to keep deformation to a minimum. Under standard operation conditions, primary slit openings that permit an overfilling of the first mirror in order to obtain a homogeneous heat load are thus recommended. An eventual deformation of the mirrors can be to some extent compensated for by the fact that the second mirror is bendable, allowing in addition pre-focusing for collimation of the beam on the monochromator or for focusing on the sample or on a secondary source situated between mirror and sample position.

#### 2.2.1. The double-crystal and channel-cut monochromator   

**Figure 3:**
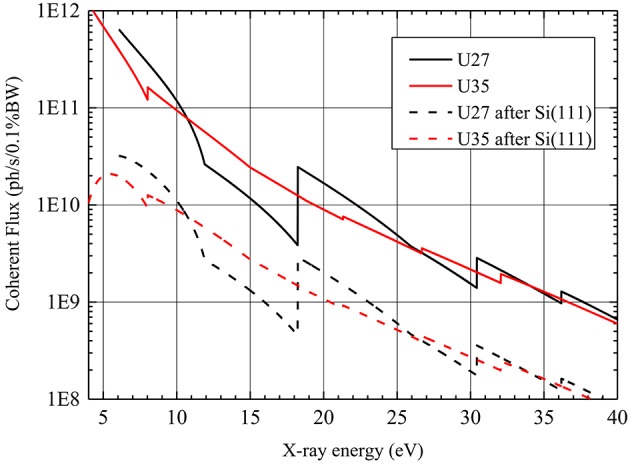
Polarization and bandwidth-corrected calculated coherent flux for the double-reflection Si(111) monochromator.

As a high-flux crystal monochromator, a Si(111) double-reflection device in non-dispersive geometry is a very common choice at hard X-ray synchrotron beamlines. For the concerned energy regime at ID01 the reflection- or Bragg geometry is best suited. For nano-diffraction, stability is one of the prime concerns when designing an instrument; an as-rigid-as-possible connection between the two crystals has to be assured in order to avoid directional fluctuations of the beam. This is best met by a channel-cut device, but comes at the expense of limited surface quality, the inability to tune the beam position and a limited energy range. All those drawbacks can be compensated for by a double-crystal design at the risk of lower stability. The ID01 monochromator was designed to marry the best of both approaches. In horizontal scattering geometry a double-crystal setup and a channel-cut device are superimposed on an in-vacuum rotation table with vertical axis. By a translation in height an interchange between both systems is possible. Both crystal monochromators are cooled by liquid nitrogen and are situated at 33 m from the X-ray source. The horizontal geometry assures highest angular stability of the setup. Differential pumping is avoided by using an in-vacuum rotation stage for the Bragg angle. Throughout the whole primary optics ion pumps suffice for normal beamline operation and thus no mechanical vibrations are introduced by mechanical pumps. The drawback of the horizontal geometry is a loss in intensity, as observed in Fig. 3 ▸, on the low-energy side of the spectrum due to the linear polarization of the synchrotron radiation in the plane of diffraction.

**Figure 4:**
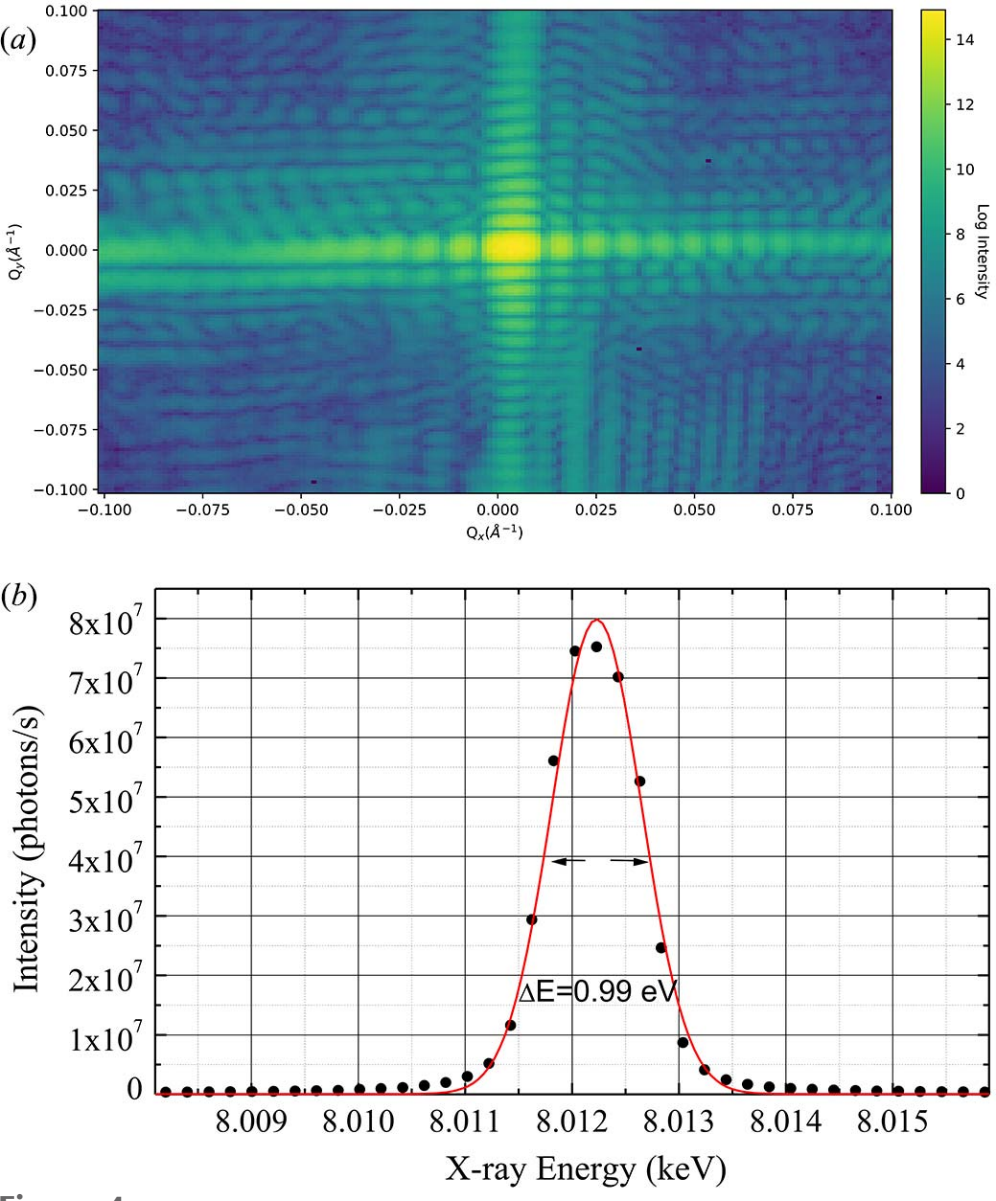
(*a*) Absolute flux measurement through a small aperture using a monochromatic beam with a Maxipix detector. (*b*) Bandpass characterization of the Si(111) monochromator at 8 keV.

The flux delivered to the endstation by the crystal monochromator was characterized with a precision slit in the beam at 100 m from the source (the secondary source slit discussed below). A Maxipix (Ponchut *et al.*, 2011 ▸) photon-counting pixel detector with 55 µm pixel size was used at a distance of 118.85 m from the source, and closing the slits to 10 µm × 10 µm a Fraunhofer diffraction pattern was recorded on the detector. The absorption of a total of 135 µm of polyimide windows and 0.51 m of air were considered. This allows measurement of the total flux with only a few filters in the beam and at the same time determination of the precise slit size by fitting it to the analytical description of the Fraunhofer pattern. The recorded pattern is shown in Fig. 4(*a*) ▸, corresponding to a real slit opening of 9.7 µm × 8.6 µm. The measured flux after correction for absorption in air gaps and windows was 8.5 × 10^5^ photons s^−1^ for a U27 undulator, of 1.6 m length. The theoretical flux emitted in the bandpass of a Si(111) monochromator can be calculated by the undulator period and the electron beam parameters of the ESRF. For 200 mA filling the expected theoretical flux amounts to 1.35 × 10^7^ photons s^−1^ µm^−2^ for a bandpass of 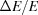 = 0.1%. Taking into account the polarization losses of 40% at 8 keV and the measured bandpass of 1.235 × 10^−4^, the delivered flux corresponds to 82% of the theoretical value. The losses are expected to originate from slight undulator errors (aberrations of the magnetic field through the length of the device) and monochromator aberrations due to deformation under heat load. The bandpass delivered by the Si(111) monochromator as measured by a Si(004) crystal is presented in Fig. 4(*b*) ▸.

#### 2.2.2. The multilayer monochromator   

**Figure 5:**
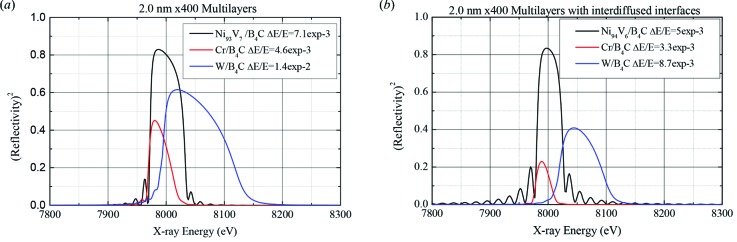
(*a*) Simulated reflectivity from ideal multilayer monochromators in the vicinity of 8 keV for different materials. (*b*) Similar situation as in (*a*) but with 0.3 nm interdiffusion length across the interfaces.

The choice of crystal monochromators available in general deliver energy resolutions of 10^−4^ or better. However, some experiments can tolerate a broader bandwidth. This is particularly relevant for the study of nanostructures where the tolerable maximum bandwidth should correspond to the inverse of the typical sample size. More precisely, it is the sample size projected to the scattering vector *Q* that is the limiting factor. The smaller this size, the higher the tolerance of the sample to a larger bandpass of the incident X-ray. This is a very important consideration, as smaller samples lack scattering power and motivate the hunt for a higher incident intensity at the expense of energy resolution. Ideally one would like to match the bandwidth selected by the primary optics to the requirement of the sample but in reality one has usually to face a resolution gap between 10^−4^ as supplied by Si(111) monochromators and a few 10^−2^ as supplied by the undulator harmonics that may be selected by total reflecting mirrors or multilayer monochromators. The use of a multilayer monochromator that significantly outperforms the typically available 2% bandwidth of undulators seems to be self evident but is technically challenging. For a high reflectivity of a multilayer one typically chooses materials with highest possible contrast, *e.g.* W and B_4_C as already implemented on the white-beam mirrors of ID01. This, however, leads to a high reflectivity at every interface so that all X-rays are reflected after only a limited amount of layers. If only high reflectivity is sought, such high-contrast materials are the first choice as they require only a low number of layers that have to be deposited identically and hence relax the requirements for the quality of the deposition. If lower-density materials are chosen, the reflectivity per interface is reduced and more interfaces can contribute to the reflection, the reflection criterion becomes more selective and thus the bandwidth may decrease. However, this requires a higher quality of growth as the required precision of the layer thickness increases as well. In a simplified manner one can define the energy resolution 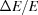 = 1/*N* with *N* being the number of efficiently illuminated layers. Consequently, in order to make the energy resolution better, one needs to choose weaker absorbing and scattering materials and increase the number of identical layers. Towards lower X-ray energies, absorption tends to be the main limitation for the number of layers that can effectively be seen. In this case the reflection curve is expected to be of Lorentzian shape corresponding to the Fourier transform of an exponentially damped wave. For higher X-ray energies extinction becomes the dominating factor leading to a dynamical behaviour of the reflectivity. With the increase in number of layers to be grown comes the natural risk of amplifying surface roughness leading to a loss in reflected peak intensity. Furthermore, to keep the constructive interference condition between all layers, the total thickness tolerance of a sandwich of *N* layers is 1/*Nd* with *d* being the multilayer period. It is thus a great challenge to grow high-resolution multilayers on large substrates. The horizontal scattering geometry and the big horizontal beam at ID01 make large-area multilayers necessary. In addition, the multilayer periodicity *d* has to be chosen to be as thin as possible to reduce the trajectory of the X-ray beam through the material. This is the most important parameter for the reduction of absorption as any reduction of *d* naturally reduces the overall thickness but also increases the Bragg angle of the multilayer and thus reduces the required length of the substrate. Typical candidate materials may be Ni or Cr to replace the often used W as a strongly reflecting material. Example calculations are shown in Fig. 5(*a*) ▸ for multilayers of 400 bilayers and a periodicity of 2.0 nm and are at the cutting edge of what can be manufactured reliably on large areas. The compared materials are Cr, Ni_93_V_7_ and W, each with B_4_C as spacer material. The obtained bandwidth is of Δ*E*/*E* = 4.6 × 10^−3^ for Cr, 7.1 × 10^−3^ for Ni_93_V_7_ and 1.4 × 10^−2^ for W. In these simulations perfectly flat interfaces have been assumed. In reality some interdiffusion takes place and from experience an interdiffusion length of 0.3 nm has been assumed for the data in Fig. 5(*b*) ▸. Interdiffusion reduces the reflectivity at each interface without affecting the absorption throughout the multilayer. As a result, the bandpasses are reduced for all materials; however, the reflectivities are severely affected for Cr and W only. Ni_93_V_7_ keeps a good peak reflectivity as extinction dominates absorption in the interdiffusion-free case. The loss of reflectivity at the interfaces is partially compensated by a contribution of more layers improving the bandpass from 7.1 × 10^−3^ down to 4.5 × 10^−3^ while reducing the peak reflectivity from 0.83 to 0.70. Ni_93_V_7_/B_4_C were thus chosen as the material of choice for 8 keV and around 20 keV (third harmonic regime of the U27 undulators). Ni_93_V_7_ was chosen here instead of Ni as it is a non-ferromagnetic alloy and thus compatible with magnetron sputter deposition.

**Figure 6:**
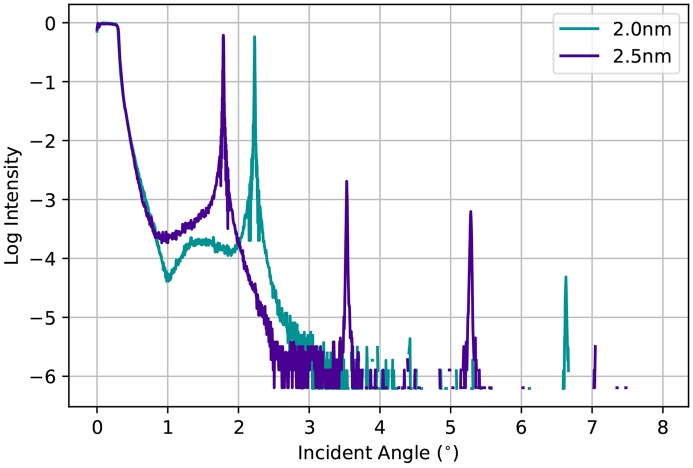
Reflectivity of the 2.0 nm and the 2.5 nm multilayer monochromator analysed with a Cu *K*α laboratory source.

The Ni_93_V_7_/B_4_C multilayers made for ID01 were deposited in two different stripes on 300 mm-long substrates (Morawe *et al.*, 2017 ▸). Two different periodicities were chosen, again in order to cover a wide energy range for a limited angular range. The periodicities are 2.0 nm and 2.5 nm, respectively. Laboratory source X-ray characterization after the manufacturing of the mirrors is presented in Fig. 6 ▸ and shows a peak reflectivity for the single multilayer mirror of 0.62 (2.0 nm) and 0.65 (2.5 nm). The resulting efficiency of the double-reflection device is 0.38 (2.0 nm) and 0.42 (2.5 nm). Compared with what could be expected from simulations, this efficiency is slightly lower. The reasons lie in a limited substrate quality (roughness) that leads to losses in the specular reflected intensity. The achieved energy resolution at 8 keV for a double-reflecting mirror is 0.31% (2.0 nm) and 0.54% (2.5 nm). These values were derived from the single reflection bandwidths determined to be 0.37% (2.0 nm) and 0.64% (2.5 nm).

#### 2.2.3. Comparison of flux obtained from the different monochromators   

**Figure 7:**
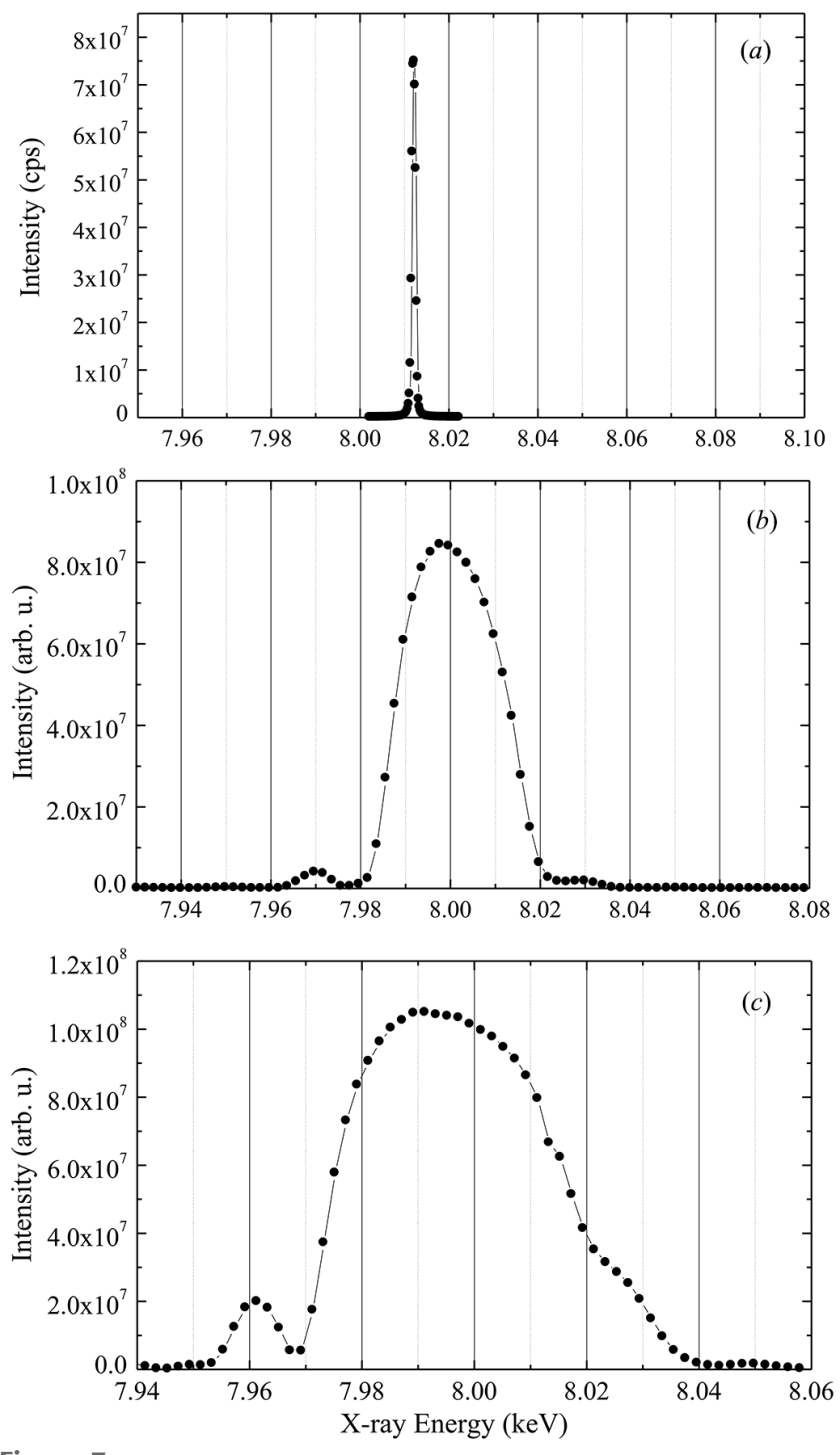
Bandpass analysis of all three monochromators with a Maxipix detector: (*a*) Si(111) double-crystal monochromator, (*b*) 2.0 nm multilayer monochromator, (*c*) 2.5 nm multilayer monochromator.

Total flux measurements from synchrotron sources allow verification of the proper functioning of all involved elements. Similar to the total flux measurement described above for the Si(111), both multilayer monochromators were tested. In addition, their bandwidth under real conditions were compared at the beamline. The monochromators were illuminated with the white synchrotron beam and aligned to the fundamental line at 8 keV from the 1.6 m U27 insertion device. Their bandpass was characterized by a Si(004) Bragg reflection on the diffractometer in the experimental hutch and the total flux was measured by analysing the integrated flux from a Fraunhofer diffraction pattern similar to the one shown in Fig. 4 ▸. The scans through the bandpass are shown in Fig. 7 ▸. The measured bandwidths are listed in Table 2 ▸, together with the observed flux values in photons s^−1^ µm^−2^ as determined for a 1.6 m device. The total usable device length of 4.4 m (3 m U27 and 1.4 m U35) supplies about a factor of 2.5 times this value. The last column contains the flux gain as compared with Si(111).

### 2.3. Secondary optical elements   

#### 2.3.1. The second optics hutch and the transfocator device   

At 55 m from the source and thus roughly half way between source and sample or between source and secondary source, two sets of Be lenses are installed. One has 2D parabolic lenses for focusing in two dimensions, one with parabolic cylinders for vertical focusing only. The latter offer a large horizontal aperture, the direction in which the beam can be focused with the white-beam mirrors. This device fulfils three main functions. First, for high-flux experiments like SAXS or microscopy it condenses a significant fraction of the undulator beam on the sample. Second, for nanobeam experiments using a secondary source the beam can be focused on a pair of slits serving as secondary source at 100 m from the primary source. Third, it can be used as a pre-focusing device in nano-focus or coherent diffraction experiments in order to find the right compromise between beam flux- and size or to match the coherence length to the aperture of the nano-focusing device.

The 1D refractive lenses are mounted in the following configuration:

(i) 15 lenses, 200 µm minimum radius of curvature (four actuators with one, two, four, eight lenses).

(ii) 15 lenses, 300 µm minimum radius of curvature (four actuators with one, two, four, eight lenses).

(iii) 15 lenses, 500 µm minimum radius of curvature (four actuators with one, two, four, eight lenses).

(iv) One lens, 1500 µm minimum radius of curvature (one actuator).

(v) One lens, 1000 µm minimum radius of curvature (one actuator).

The 2D refractive lenses are mounted in the following configuration:

(i) 15 lenses, 500 µm minimum radius of curvature (four actuators with one, two, four, eight lenses lenses).

(ii) One lens, 1500 µm minimum radius of curvature (one actuator).

(iii) One lens, 1000 µm minimum radius of curvature (one actuator).

#### 2.3.2. The secondary source on the ‘golden’ slab   

In order to anticipate potential instabilities in the primary optics or mutual movements between source and endstation that are on different concrete slabs, a pair of high-precision slits can serve as a virtual source at 100 m from the primary source. It is situated in a small third optics hutch and at this position it is still 20 m from the sample and is positioned on the same concrete slab. This slab was conceived considering highest vibrational and thermal stability. As a variable aperture this device allows for an easy tuning of the compromise between beam spot size on the sample and beam flux without realignment of the nano-focusing optics. The secondary source also allows the coherence lengths to be optimized for each experiment (Robinson, 2008 ▸). At the time of writing, the secondary source has not been fully exploited. The second white-beam mirror which focuses the beam, in the horizontal, onto the secondary source is not stable enough to execute coherence-based experiments.

## 3. Nanofocus endstation   

**Figure 8:**
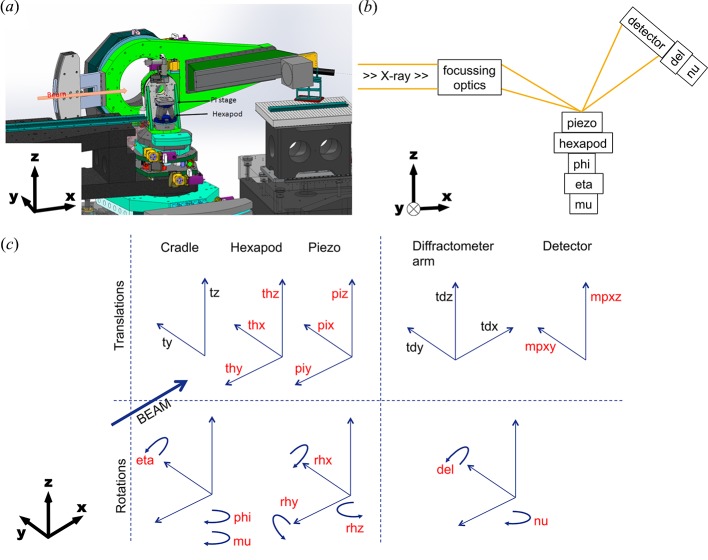
Computer-aided design image (*a*) and schematic (*b*) of the diffractometer and sample stack at the nano-focus endstation. (*c*) All available sample degrees of freedom (red: typical for user experiments).

The nanofocus endstation naturally delivers stability significantly smaller than the size of the beam itself whilst providing the flexibility to manipulate samples through both large distances and large angles with accuracy and precision relative to the reference laboratory frame, shown in Fig. 8(*a*) ▸.

The reference frame for the beamline is: positive *x* along the beam direction, positive *y* outboard of the synchrotron and positive *z* upwards. The nanofocus endstation consists of a large granite table on which both the focusing optic and the sample environment sit. The available focusing optics are Fresnel zone plates (FZPs), Kirkpatrick–Baez (KB) mirrors or compound refractive lenses. Typically the beam incident on the focusing optic is defined by a slit assembly (W cylinders), with an intensity monitor (silicon photodiode measuring scatter from a polyimide window) immediately behind providing the reference beam intensity (I_zero_). The FZPs available have 300/200/120 µm diameter with 70 nm/60 nm outer zone width or 200 µm diameter with 45 nm outer zone width (Leake *et al.*, 2017 ▸). The KB mirror has a 200 µm × 176 µm clear aperture, the vertical mirror at 230 mm and horizontal mirror at 133 mm focal distance. Both focusing optics operate in a flowing nitrogen atmosphere. Typical flux and focal spot sizes are shown in Table 3 ▸.

The sample manipulation, depicted in Fig. 8(*b*) ▸, consists of four categories of motor, starting at the bottom. First, a *z*-translation controlled by a tripod of motors and a *y*-translation; second, three Huber circles of rotation, denoted mu [left-handed (LH) rotation around *z* axis], eta (LH rotation around *y*) and phi (LH around *z* when eta is zero); third, a Symmetrie BORA hexapod with three axes of translation with right-handed rotation for each axis; and finally a PI-Mars three-axis piezo (100/100/20, 200/200/200 µm stroke in *x*, *y* and *z*, respectively). The sphere of confusion for the three sample circles is <10 µm. The sphere of confusion of the hexapod is <50 µm and currently the limiting factor to sample alignment. The piezo motors provide a few nanometeres resolution. The top plate of the piezo sits 50 mm from the centre of rotation, allowing ample room for the sample environments up to a few kilograms in weight.

The detector arm is decoupled from the optics and sample, with two left-handed rotation axes, denoted nu and delta, around *z* and *y*, respectively. A Maxipix detector (55 µm × 55 µm pixels, 516 × 516) (Ponchut *et al.*, 2011 ▸)/Eiger detector (75 µm × 75 µm pixels, 1030 × 2164) is placed on the arm, with limits of up to 2.36 m to delta 68° and 1.76 m to delta 120°. The angular stroke of all rotation axes in degrees are: −10 < nu < 110, −3 < delta < 120, −100 < phi < 100, −1 < eta < 95, −5 < mu < 90. All available sample degrees of freedom are shown in Fig. 8(*c*) ▸.

The hutch temperature is closely regulated to ±0.07°C. Upon closing the door typical drift values of the order of 60–100 nm per hour are observed; ultimate stability is often reached after several days without intervention. Vibrations are significantly smaller than the beam size, reflecting the focused X-ray beam from a silicon wedge, whose projection is smaller than the beam size, showing intensity fluctuations of the order of ±5% which corresponds to ∼20 nm.

At present several environment chambers and sample mounts are available:

(i) Furnace, 8 mm-diameter Eurotherm-controlled ceramic heater up to 1000°C, 50°C min^−1^ vacuum or gas environment (Richard *et al.*, 2017 ▸).

(ii) Kinematic mounts for reproducible sample positioning both in air, rough vacuum or gas environments with a polyimide dome.

(iii) Electrochemistry cell, allowing a thin layer of electrolyte to be confined between a polyimide window and a substrate.

(iv) A continuous-flow mini-cryostat capable of 3 K with electrical feedthroughs for additional characterization.

(v) Portable set-ups designed by the user community can be easily accommodated, such as a nanocrystal indenter (Ren *et al.*, 2014 ▸) or a biaxial deformation rig (Van Petegem *et al.*, 2017 ▸).

### 3.1. Beam-focusing modes   

**Figure 9:**
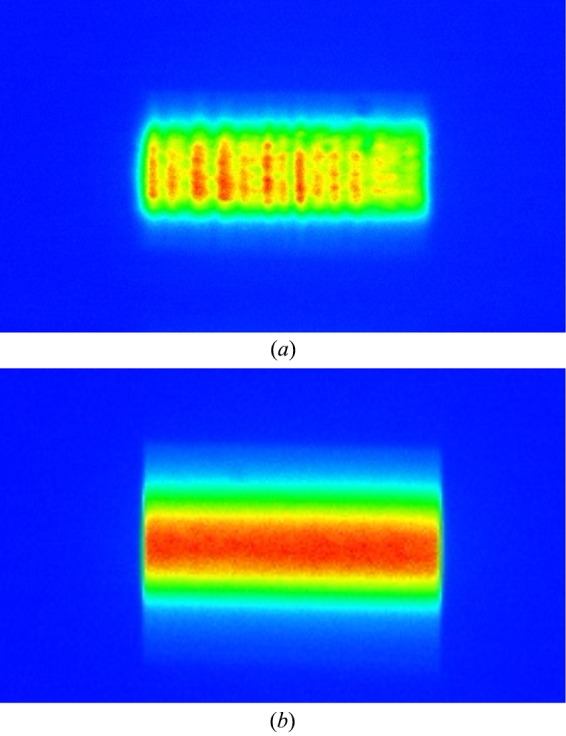
Typical wavefronts 100 m from the source. (*a*) Channel-cut monochromator and (*b*) double-crystal monochromator. Dimensions: 2 mm × 5 mm (V × H).

The different focusing modes and monochromator modes are specified for their anticipated applications. The specifics of the optic components upstream of the experimental endstation have been covered previously. To summarize: a white-beam mirror can be employed to reduce heat load on the monochromator of choice and apply pre-focusing when required, reject higher harmonics but may be bypassed if optimal wavefront preservation is required. A channel-cut silicon crystal monochromator is available for the most stable incident beam, the disadvantage being that the polishing of the surface is not ideal and therefore for wavefront-sensitive techniques a double-crystal monochromator is preferred, see Fig. 9 ▸. These options incur a natural bandwidth of 10^−4^, the alternative is to employ a multilayer mirror; at the expense of bandwidth 4.5 × 10^−3^, a factor of 20 gain in flux is achieved in certain scenarios, again sacrificing the quality of the wavefront of the incident beam. Example wavefronts are demonstrated later in Fig. 13.

The achieved demagnification of the source is the ratio between the source–optic and optic–sample distances, hence for the smallest beams the optic must be placed as close to the sample as possible but is fundamentally limited by the numerical aperture or quality of the optic itself and technically by the space required for the sample environment. For nano-focused beams either FZPs or KBs are available; the beam sizes, delivered photon flux and working distances are defined in Table 3 ▸. For micro-focus beams (1–2 µm), compound refractive lenses (CRLs) are used.

Alternatively, for larger beams at the sample position the white-beam mirror and vertical transfocator are employed to focus the beam to the sample position and deliver a beam of typically 25 µm × 140 µm; inevitably the angle of incidence of the chosen Bragg reflection will spread the beam out in the horizontal direction, allowing single-shot full-field diffraction imaging when an optic is placed after the sample and magnifies the diffracted beam by approximately a factor of 65.

### 3.2. Available techniques   

The techniques available at ID01 include: highly focused beams for nano-diffraction mapping and coherent diffraction imaging (CDI), full-field diffraction microscopy, grazing-incidence diffraction and small-angle scattering. The principle techniques will be described in depth.

#### 3.2.1. Nano-diffraction   

**Figure 10:**
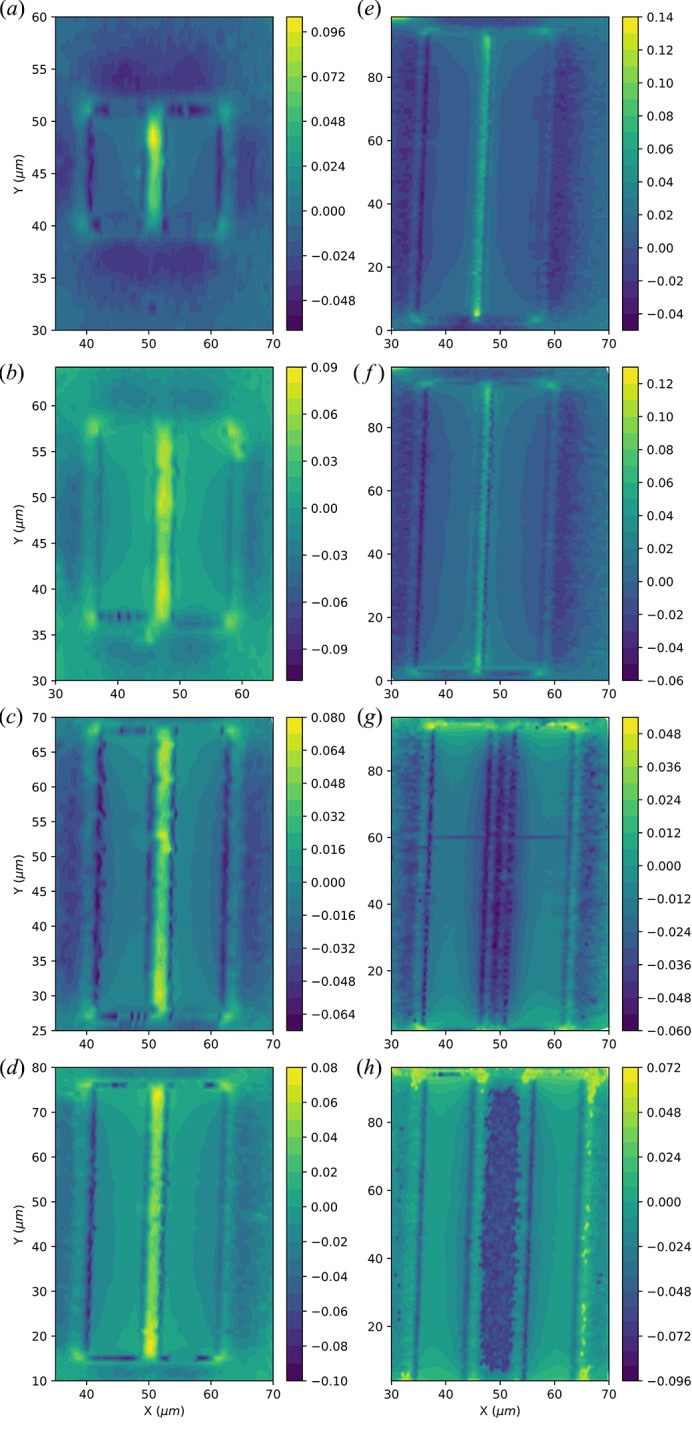
Exhaustive study of two-dimensional out-of plane strain component ∊_*zz*_ (%) distribution over Ge microstripes of different dimensions: (*a*)–(*e*) for a width *w* = 2 µm and a length *l* = 10, 20, 40, 60 and 90 µm, respectively, and (*f*)–(*h*) for a width *l* = 90 µm and *w* = 1, 2, 5, 10 µm, respectively. With a resolution of 0.5 µm, an acquisition time of 30 ms per detector image, these samples were measured and analysed within three days.

The hardware components available at the beamline made possible the development of a continuous quick mapping scanning mode (Kmap) (Chahine *et al.*, 2014 ▸). In this mode, linear, or two-dimensional (2D), continuous scans are made with one, or two, of the three encoded piezoelectric stack motors on which the sample is mounted. During these scans, the motors move continuously with 2 nm spatial resolution and over a range up to 200 µm. Simultaneously, the fast photon-counting detector acquires 2D images at a frequency synchronized with the motor movements. These tasks are orchestrated by a synchronization card (MUSST) developed at the ESRF. In this approach the *SPEC* software, routinely used to control the hardware components, is replaced by the MUSST, reducing considerably the overhead on each point. This allows fast non-destructive direct imaging under diffraction conditions with a nanofocused X-ray beam of thin layers (Richard *et al.*, 2015 ▸), heterogeneous devices (Vianne *et al.*, 2015 ▸), microstructures (Chahine *et al.*, 2015 ▸), nanoparticles and nanowires of semiconductor devices, micro-grains in polycrystalline structures for photovoltaic devices (Schäfer *et al.*, 2016 ▸) or even macro-grains and grain boundaries in heterogeneous metallurgy alloys (Filippelli *et al.*, 2016 ▸). The benefits of the Kmap technique are twofold: (i) features may now be quickly identified using diffraction contrast and localized, thus saving precious time for the experiment, (ii) reciprocal-space maps can be built up over two-dimensional regions of the sample within a few hours instead of several days. Reciprocal-space maps are acquired by repeating Kmap scans for several incident X-ray angles covering the range of a typical rocking curve of a Bragg peak, adding a third dimension to the already 2D detector data. The location and shape of the Bragg peak in reciprocal space can now be extracted for each direct-space position on the sample, thus tracing landscapes of local structural variations with a direct-space resolution defined by the beam size. This is done by using the *Strain and Orientation Calculation Software* (*XSOCS*) (Chahine *et al.*, 2014 ▸) developed at the beamline in order to handle, and make an online treatment of, millions of two-dimensional detector images. The resulting five-dimensional generated datasets are mined using an easy-to-use GUI interface to the *XSOCS* software package (https://gitlab.esrf.fr/kmap/xsocs.git). A three-dimensional visualization tool can be used to select a volume of interest in reciprocal space, therefore isolating a specific material of a strained/tilted region, on which data treatment will be performed. The result is a quantitative determination of strain, tilt, thickness fluctuations and composition maps. Variations of strain (10^−5^ relative lattice variation) and tilts (10^−3^ degrees) are visible, well below the instrument resolution, as the relative shift, shape and intensity of the Bragg peak are analysed when moving from one position to another on the sample. With many sample environments available at the beamline compatible with Kmap, we now offer the possibility to conduct *in situ* and *operando* measurements on samples of high technological relevance to understand the effects of external constraints such as electric fields, temperature, mechanical deformation and geometrical dimensions (see Fig. 10 ▸) on the structural parameters and the physical properties.

#### 3.2.2. Full-field diffraction X-ray microscopy   

**Figure 11:**
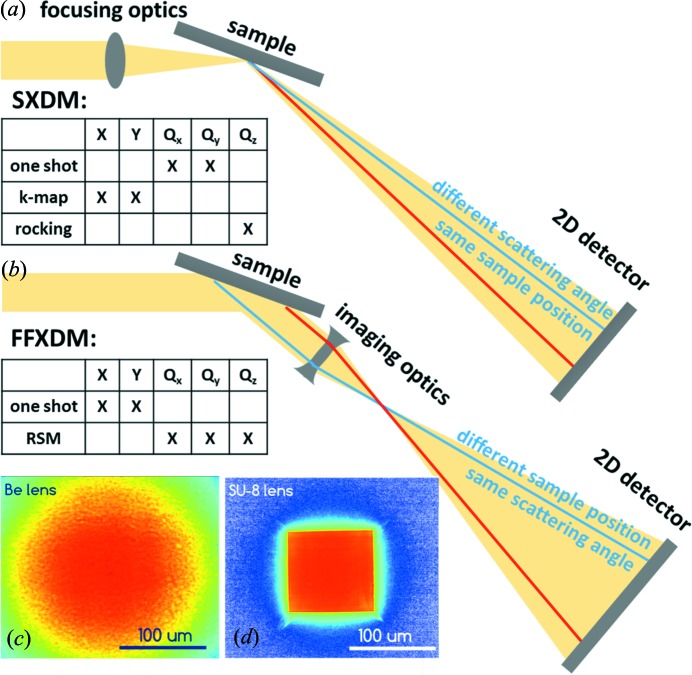
Schematic of (*a*) SXDM versus (*b*) FFDXM in terms of direct- and reciprocal-space dimensions probed and time required. (*c*) Beam from Be CRLs and (*d*) beam from SU-8 CRLs measured with FFDXM.

Full-field diffraction X-ray microscopy (FFDXM) is a novel technique currently being developed at ID01 (Hilhorst *et al.*, 2014 ▸) and elsewhere (Simons *et al.*, 2015 ▸; Laanait *et al.*, 2014 ▸). It can be used to acquire the same information as a scanning X-ray diffraction microscope (SXDM), but via a different concept. In SXDM [see Fig. 11(*a*) ▸], a set of focusing optics is placed upstream of the sample to achieve a nano-size beam. With one single acquisition, different pixels on the 2D detector measure scattered photons from the same sample position but at slightly different exit angles (*i.e.* 0D resolution in direct space, 2D in reciprocal space). Additional information in direct space is achieved, similar to the Kmap method, by raster scanning the sample. In FFDXM [see Fig. 11(*b*) ▸], a set of objective optics (essentially the same as those used otherwise for focusing) is placed downstream of the sample to provide a magnified image of the scattered beam. With a single acquisition, different pixels on the 2D detector measure scattered photons from the same exit angle but at different sample positions (*i.e.* 2D resolution in direct space, 0D in reciprocal space). A quasi parallel incident beam is required in this case to maximize the tilt and strain resolution, which explains the absence of focusing optics in the setup. Additional information in reciprocal space is achieved, as in the case of having a point detector, by reciprocal-space mapping (RSM) with the diffractometer.

ID01 offers several imaging lens options (see Table 4 ▸) tailored to each experiment. The Be CRLs (50 lenses of 50 µm radius at parabola apex) [see Fig. 11(*c*) ▸] is the standard optic at 8 keV, and can be used in principle at any given energy (Snigirev *et al.*, 1996 ▸). However, above 20 keV its focal distance is too long, resulting in insufficient magnification ratio and numerical aperture for proper imaging. The SU-8 CRL (100 lenses of 6 µm radius at parabola apex) is the standard optic for higher energies and can be used up to the Sb *K*-edge at 30.5 keV (Marschall *et al.*, 2016 ▸). At 19.5 keV, the SU-8 lens offers a rather similar characteristic (focal distance, resolution and transmission) as the Be lenses at 8 keV [Fig. 11(*d*) ▸]. Its smaller effective aperture *D*_eff_ is made up for by the homogeneous background produced by its polymer lens elements. For those in need of a better spatial resolution, multilayer Laue lenses (MLLs) can be used instead (Niese *et al.*, 2014 ▸). The MLL can achieve sub-50 nm resolution, better efficiency and overall a higher numerical aperture at the cost of a shorter working distance and a smaller field of view.

**Figure 12:**
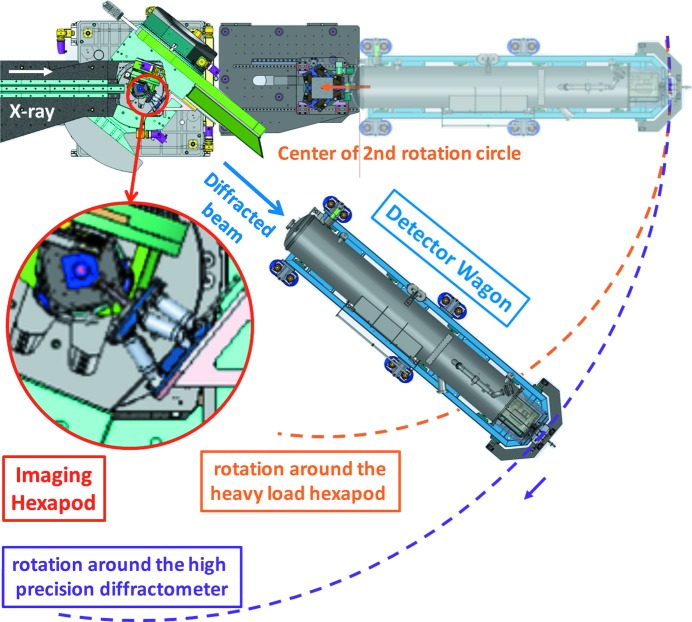
Birds-eye view of the experiment hutch. The detector tube can rotate around two endstations, nano or heavy load. Inset: a zoom of the FFDXM lens mount.

The lenses are held on a hexapod mounted on the diffractometer arm; alignment is achieved by three hexapod translations and three rotations (see Fig. 12 ▸). For the SU-8 lenses (optional for MLLs), an 80 µm (25 µm in the case of the MLL) aperture is added with adjustable distance to the lenses. The alignment of the aperture is achieved by two Smaract translation stages. The detector is an Andor Zyla 5.5 sCMOS camera with 5M (2560 × 2160) pixels, sitting on the detector wagon. The wagon can move freely inside the gigantic vacuum pipe (10^−2^ mbar) to achieve a lens–detector distance of 3.0–6.5 m. The physical size of each pixel is 6.5 µm × 6.5 µm. At 6.5 m distance and with a lens of 100 mm focal length, this is equivalent to a magnification ratio of 65× and an effective pixel size of 100 nm. The Andor camera is fibre coupled to a 15 µm of Gadox scintillator for the optimal efficiency and low afterglow. Better resolution can be obtained at the cost of intensity and significant afterglow by switching to a 20 µm-thick LuAG scintillator. Occasionally a 25.7 µm-pitch Scint-X structured scintillator is used for higher energies. By default, the vacuum pipe (and hence the detector) rotates around the centre of rotation (CoR) of the Huber diffractometer. As a result, the sample is usually mounted vertically to allow the scattering signal to be measured in the horizontal plane. Alternatively, the vacuum pipe can be reconfigured to rotate around the CoR of the heavy-duty hexapod. The heavy-duty hexapod allows for a more complex sample environment to be installed (maximum load: 50 kg), and is situated 2.034 m downstream of the diffractometer.

For experiments involving weak deformation in perfect crystals, the DCM is in most cases mandatory as it gives the best strain and tilt resolution. For experiments involving thin film or nanocrystals, the 20× flux increase of the multilayer monochromator (MM) outweighs its poor energy resolution. In either case, transfocators are used to bring in as many photons as possible onto the sample while keeping the incident beam quasi-parallel. Despite being capable of delivering the same information as the Kmap, the FFDXM is in fact more suited for *in situ* and *operando* experiments. The standard exposure time is of the order of 0.1–10 s, and a rocking curve of an entire 200 µm × 200 µm surface can be measured in a matter of minutes.

#### 3.2.3. Coherent diffractive imaging   

The Kmap technique provides a large field of view with a spatial resolution defined by the beam size, allowing both strain and tilt to be decoupled, and requires several hours for acquisition. The FFDXM method offers, with sub-second exposure times, a similar field of view and similar resolution defined by the optic as the Kmap; however, it is not possible to decouple strain and tilt in a single acquisition. Improved X-ray optics are difficult to realize; an elegant solution is to remove the need for the optic by exploiting the coherence of the incident X-ray beam. Retrieval of the phase information lost during the measurement, of the interference pattern from a coherent beam and a scattering object, allows one to obtain a resolution independent of the beam size which depends upon the spatial extent of the scattered intensity in reciprocal space, fundamentally limited by the wavelength of the incident light. When applied to Bragg diffraction peaks the direct-space resolution exceeds 5 nm or better but the strain resolution can enter the realm of a few picometres (Robinson *et al.*, 2001 ▸).

The source size and its distance from the focusing optic define the coherence lengths and thus the typical aperture placed before the optic, 396 µm in the vertical and 79.2 µm in the horizontal. As the apertures of the optics are relatively small (200/300 µm FZP and 200 µm KB), it can be advantageous to pre-focus and boost the coherent flux in the experiment. In addition it is preferable to use a more symmetric beam shape, dependent on the incident angle of the experiment and the sample itself; typically a 200 µm × 60 µm aperture is used. For FZPs the aperture is offset to reduce the impact of the central stop (required to block the zeroth-order light) so a larger aperture, 300 µm, produces a more asymmetric beam shape due to the partial illumination of the edges of the FZP without a large gain in flux. Typical beam sizes are of the order of several hundred nanometres, see Table 3 ▸, with a longitudinal coherence length of 800 nm, set by the monochromator (Leake *et al.*, 2009 ▸).

**Figure 13:**
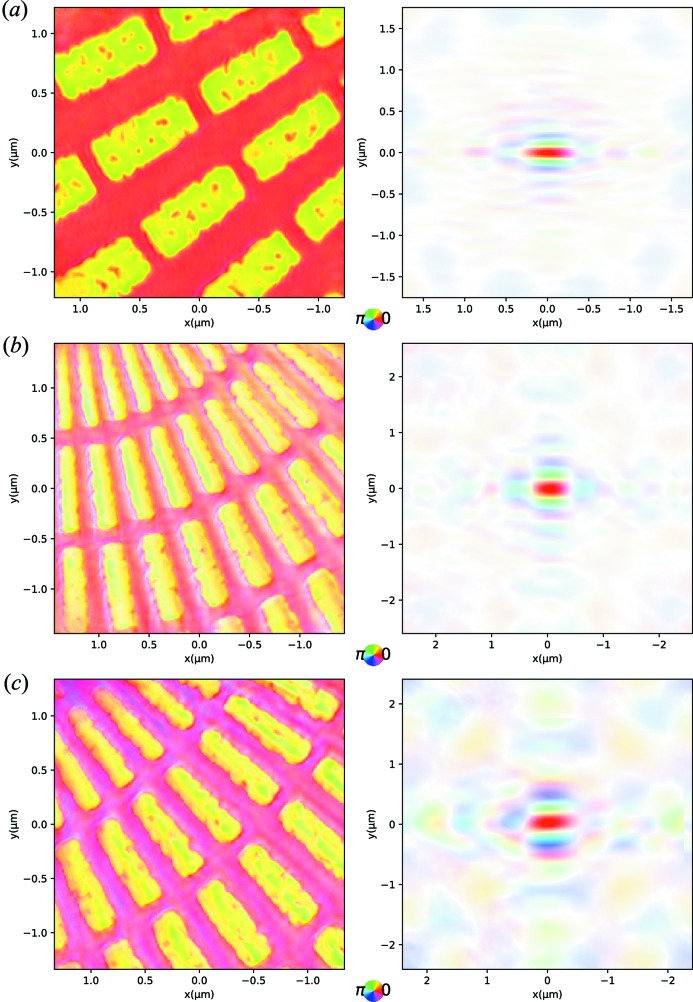
Typical reference object (left column) and probe (right column) reconstructions using ptychography for (*a*) double-crystal monochromator + Fresnel zone plate, (*b*) double-crystal monochromator + KB mirror and (*c*) multilayer monochromator + KB mirror reconstructed using the *PyNX* package. Brightness represents the amplitude and colour the phase of the image. The resolution for reconstructions (*b*) and (*c*) is lower than (*a*) due to the reduced numerical aperture of the KB, compared with the Fresnel zone plate, which necessitates attenuators to be used to protect the detector.

**Figure 14:**
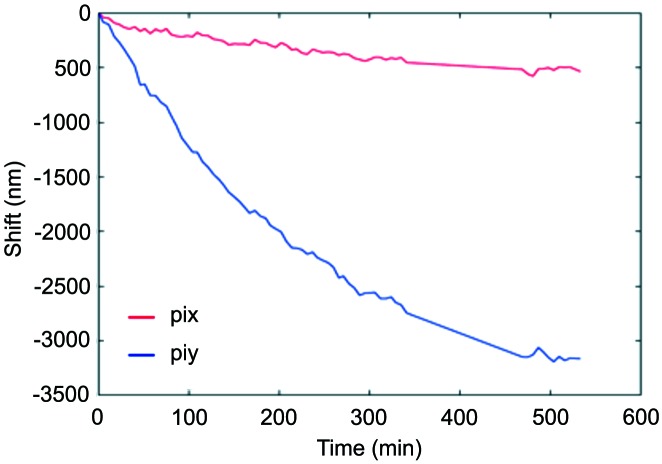
Shift of a Kmap determined via sub-pixel cross-correlation for a 2D map of the same sample region over a 9 h period. ‘pix’ and ‘piy’ correspond to the *x*- and *y*-translation, respectively, of the piezomotors located beneath the sample.

The success of a CDI experiment is dependent on the quality of the X-ray beam; the wavefront should remain constant for the duration of the experiment. In general, the number of components in the beam are kept to an absolute minimum, the use of windows is limited to those required to bridge ultrahigh-vacuum/vacuum and vacuum/air transitions in the experiment’s hutch. To ensure this, for each experiment the beam incident on the sample is fully characterized using ptychography of a reference sample (Siemens Star) (Pfeiffer, 2018 ▸), and can be repeated in as little as 30 min at any time during the experiment; see Fig. 13 ▸. In general, a small primary slit (0.5 mm × 0.5 mm) is used to reduce both the heat load on the monochromator and the settling time of the monochromator, particularly relevant for significant top-ups in the synchrotron beam. The high-quality Si double-crystal monochromator produces a very flat illumination over a large area, far greater than the coherence lengths, thus small movements can be tolerated as long as they are monitored with an I_zero_. With nanobeams, emphasis is placed on the stability of the sample itself. It has been measured to be of the order of 60–100 nm h^−1^ after immediate exit from the hutch, where ultimate stability is achieved after half to several days; see Fig. 14 ▸. Vibrations are known to be significantly smaller than the beam size which can be as small as 56 nm (Leake *et al.*, 2017 ▸).

**Figure 15:**
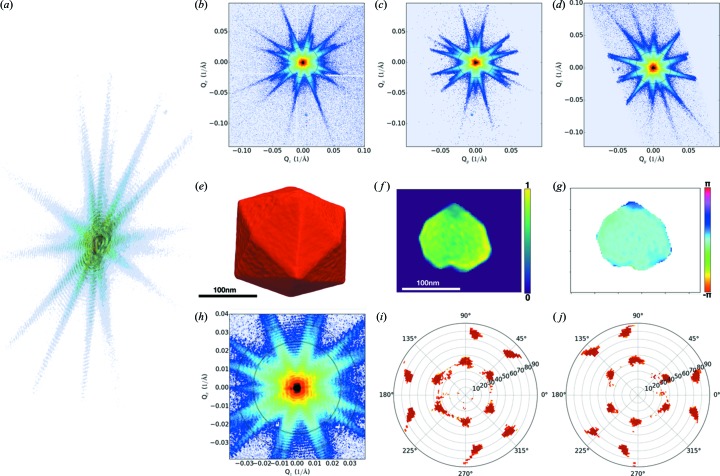
Platinum nanocrystal. (*a*) 3D render of diffraction pattern. (*b*)–(*d*) Projections of the diffraction pattern along *Q*_*y*_, *Q*_*z*_ and *Q*_*x*_, respectively. (*e*) 3D render of the reconstructed amplitudes. (*f*) Amplitude and (*g*) phase from a 2D slice through the crystal. (*h*) Zoom of (*b*), dashed circles enclose the data used to generate pole figures, (*i*) north and (*j*) south.

Fig. 15 ▸ demonstrates the typical output from online data reduction tools provided by the ID01 beamline (https://gitlab.esrf.fr/opid01/id01sware.git) and the ESRF (*PyNX*: http://ftp.esrf.fr/pub/scisoft/PyNX/doc/; *Silx Toolkit*: https://github.com/silx-kit/silx/tree/v0.9.0). An example CDI reconstruction of a Pt nanocrystal (Férnandez *et al.*, 2019 ▸) is shown in Figs. 15(*e*)–15(*g*) ▸. The dataset was obtained in 10 min (50% exposure, 1 s per point of the rocking curve) and was reconstructed with the *PyNX* software package (Mandula *et al.*, 2016 ▸). The crystal dimensions were approximately 250 nm × 250 nm × 250 nm, the photon flux incident on the sample was 1 × 10^9^ photons s^−1^ in a 400 nm focal spot. Distinct faceting was observed; the internal structure is uniform in amplitude and shows a small phase modulation close to the facets. The phasing procedure for this dataset is the standard provided by the *PyNX* package, 600 iterations of the relaxed averaged alternating reflections (RAAR) algorithm (Luke, 2005 ▸) followed by 50 iterations of the error reduction (ER) algorithm (Gerchberg & Saxton, 1972 ▸; Fienup, 1978 ▸); the support was updated every 50 iterations using the Shrinkwrap approach (Marchesini, 2007 ▸). The pole figures generated from the raw diffraction data clearly distinguish the family of {210} facets (Richard *et al.*, 2018 ▸).

#### 3.2.4. Ambition   

The design of ID01 anticipates the Extremely Brilliant Source (EBS) to be delivered at the ESRF by the middle of 2020. The biggest gains will be seen in the coherent fraction of the beam which is supposed to improve by a factor of 40. In addition to the source improvements, the optimization of undulators, bandpasses of multilayer monochromators and the less stringent size constraints of beam-tuning devices due to the smaller beam size in the optics hutches will provide several orders of magnitude of flux gain at the beamlines’ typical 8 keV operating energy depending on the desired longitudinal coherence (energy bandwidth of the monochromator). This provides access to elemental absorption edges up to 30 keV with the same experimental throughput available at the time of writing at 8 keV. It is anticipated that interferometric alignment systems will be required to take sample location to the sub-beam size level and further thermal gradient reductions arising from the miniaturization and development of new sample environments. Slight reductions in beam size and improved beam stability will be achieved through the exploitation of the secondary source and improved focusing optics. In addition, the realization of a fast Eiger detector up to 8 kHz will facilitate the acceleration of the nanofocus methods and improve the resolution of CDI datasets courtesy of a larger field of view.

## 4. Concluding remarks   

Combining full-field imaging, scanning probe imaging and coherent reconstruction methods with Bragg diffraction, the ID01 beamline at the ESRF offers a state-of-the-art strain- and structural microscope to a vast user community. Even if all three methods can be described as strain microscopy in a wider sense, they differ significantly in terms of resolution, speed, experimental boundary conditions and challenges in data treatment. Depending on the sample and questions to be answered, images of structure or strain distribution can be obtained using scanning diffraction techniques. Brought to maturity during the first phase of the ESRF upgrade these techniques allow for strain and texture imaging in thin films with a spatial resolution of 50 nm and strain sensitivity of *a*/*a* < 10^−5^. With the capacity of imaging buried layers and the enormous gain in data recording speed (becoming comparable with other scanning probe methods) this strain imaging technique offers a highly promising method for the characterization of advanced materials and devices. It is fully available and open to non-expert users and industrial clients. In terms of resolution it finds some equivalence with the full-field techniques where the optics are used after the sample rather than focusing, imaging the Bragg scattered signal on a 2D detector. The benefit of this dark-field type of diffraction full-field microscopy is to record images without sample motion. The most brilliance-dependent methods, coherent reconstruction techniques, overcome the diffraction limit of the X-ray optics as fundamental resolution limits of the first two techniques. Coherent reconstruction can be used at ID01 in order to resolve strain and structure in 3D and with spatial resolution below 10 nm. The limitations and challenges encountered here necessitate consideration of all beamline parts from primary optics, windows, focusing optics, diffractometer and detectors. These considerations have been at the origin of the beamline project ID01. A further boost of these brilliance-hungry techniques can be expected with the creation of the Extremely Brilliant Source (EBS) project from 2020 onwards.

## Figures and Tables

**Table 1:** Cut-off energies *E*_C_ in keV as a function of angle of incidence of the white-beam mirrors for the total reflection mirror stripes and Bragg energy for the multilayer stripe

Angle (mrad)	Silicon	Rhodium	Platinum	W/B_4_C (peak)
2.5	12.4	26.8	33.6	43–47
4	7.8	16.8	21.1	26–27.5
5.5	5.6	12.2	15.3	19.5–20.5

**Table 2:** Comparison of the three different monochromators at ID01: bandpass, photon flux µm^−2^, and flux compared with Si(111) monochromator at an X-ray energy of 8 keV

Monochromator	Δ*E*/*E*	Photons s^−1^ µm^−2^	Flux/Si(111)
Si(111)	1.24 × 10^−4^	8.5 × 10^5^	1
2.0 nm ML	3.2 × 10^−3^	1.47 × 10^7^	17
2.5 nm ML	5.4 × 10^−3^	3.0 × 10^7^	35

**Table 3:** Typical beam parameters at ID01 DCM = double-crystal monochromator, MM = multilayer monochromator, FZP = Fresnel zone plates, KB = KB mirrors, TF = transfocator.

Optics	Aperture (V × H) (µm)	Working distance (mm)	Beam size (µm)	Flux (photons s^−1^)
DCM + FZP	200 × 200	20/6	0.100 × 0.150	5 × 10^9^
			0.055 × 0.064	
DCM + FZP	200 × 60	6	0.064 × 0.141	2 × 10^9^
DCM + KB	200 × 200	70	0.15 × 0.24	1 × 10^10^
DCM + KB	200 × 60	70	0.15 × 0.4	4 × 10^9^
MM + KB	200 × 200	70	0.25 × 0.3	4 × 10^10^
MM + TF	200 × 200	≫10000	>25 × 120	1 × 10^12^

**Table 4:** Available objective lenses for FFDXM on ID01

	Be CRLs (ESRF)		SU-8 CRLs (KIT)		Wedged MLL (Dresden)	
*E* (keV)	8	19.5	8	19.5	8	19.5
*f* (mm)	100	400	–	100 (200)	6 (30)	14 (70)
*D*_eff_ (µm)	230	325	–	100 (200)	50 (100)	50 (100)
σ (nm)	100–200	200–300	–	100–200	50 (70)	50 (70)
*T* (%)	10	30	–	13	40 (40)	70 (70)
